# Automated analysis and detection of epileptic seizures in video recordings using artificial intelligence

**DOI:** 10.3389/fninf.2024.1324981

**Published:** 2024-03-15

**Authors:** Pragya Rai, Andrew Knight, Matias Hiillos, Csaba Kertész, Elizabeth Morales, Daniella Terney, Sidsel Armand Larsen, Tim Østerkjerhuus, Jukka Peltola, Sándor Beniczky

**Affiliations:** ^1^Neuro Event Labs, Tampere, Finland; ^2^Department of Medicine and Health Technology, Tampere University, Tampere, Finland; ^3^Department of Clinical Neurophysiology, Danish Epilepsy Centre, Dianalund, Denmark; ^4^Department of Clinical Neurophysiology, Aarhus University Hospital, Aarhus, Denmark; ^5^Department of Neurology, Tampere University Hospital, Tampere, Finland; ^6^Department of Clinical Medicine, Aarhus University, Aarhus, Denmark

**Keywords:** seizure detection, epilepsy, motor seizures, biomarkers, signal processing, artificial intelligence

## Abstract

**Introduction:**

Automated seizure detection promises to aid in the prevention of SUDEP and improve the quality of care by assisting in epilepsy diagnosis and treatment adjustment.

**Methods:**

In this phase 2 exploratory study, the performance of a contactless, marker-free, video-based motor seizure detection system is assessed, considering video recordings of patients (age 0–80 years), in terms of sensitivity, specificity, and Receiver Operating Characteristic (ROC) curves, with respect to video-electroencephalographic monitoring (VEM) as the medical gold standard. Detection performances of five categories of motor epileptic seizures (tonic–clonic, hyperkinetic, tonic, unclassified motor, automatisms) and psychogenic non-epileptic seizures (PNES) with a motor behavioral component lasting for >10 s were assessed independently at different detection thresholds (rather than as a categorical classification problem). A total of 230 patients were recruited in the study, of which 334 in-scope (>10 s) motor seizures (out of 1,114 total seizures) were identified by VEM reported from 81 patients. We analyzed both daytime and nocturnal recordings. The control threshold was evaluated at a range of values to compare the sensitivity (*n* = 81 subjects with seizures) and false detection rate (FDR) (*n* = all 230 subjects).

**Results:**

At optimal thresholds, the performance of seizure groups in terms of sensitivity (CI) and FDR/h (CI): tonic–clonic- 95.2% (82.4, 100%); 0.09 (0.077, 0.103), hyperkinetic- 92.9% (68.5, 98.7%); 0.64 (0.59, 0.69), tonic- 78.3% (64.4, 87.7%); 5.87 (5.51, 6.23), automatism- 86.7% (73.5, 97.7%); 3.34 (3.12, 3.58), unclassified motor seizures- 78% (65.4, 90.4%); 4.81 (4.50, 5.14), and PNES- 97.7% (97.7, 100%); 1.73 (1.61, 1.86). A generic threshold recommended for all motor seizures under study asserted 88% sensitivity and 6.48 FDR/h.

**Discussion:**

These results indicate an achievable performance for major motor seizure detection that is clinically applicable for use as a seizure screening solution in diagnostic workflows.

## 1 Introduction

Up to 10% of the world’s population experience at least one seizure during their lifetime (Gavvala and Schuele, 2016), and active epilepsy has an estimated 0.64% global point prevalence (Fiest et al., 2017; Beghi et al., 2019). Moreover, one-third of epilepsy patients have drug-resistant epilepsy (DRE), defined as the continuation of seizures despite using two or more anti-seizure medications (ASMs) with adequate doses either sequentially or in combination (Kwan et al., 2010). DRE is responsible for significant mortality and morbidity (Laxer et al., 2014), and the risk of premature death due to epilepsy is 11-fold in comparison to the age-matched general population or siblings unaffected by epilepsy (Fazel et al., 2013). Nocturnal motor seizures are often unwitnessed and represent a major risk factor for sudden unexpected death in epilepsy patients (SUDEP), particularly when devoid of nocturnal surveillance (Sveinsson et al., 2020). The gold standard of detecting seizures objectively, video-electroencephalographic monitoring (VEM), has high cost implications and limited access. The conventional seizure recording strategy relies on patient diaries, which have been shown to be inconsistent and unreliable, as patients significantly under-report seizure occurrence (Hoppe et al., 2007; Naganur et al., 2019). Seizure underreporting has been linked to postictal seizure unawareness and not simply the patient’s careless documentation (Hoppe et al., 2007).

While postictal interventions such as stimulation, repositioning, or airway clearing have been documented to be protective against SUDEP (Surges et al., 2009), the need for increased patient safety is still warranted and met by automated seizure detection and frequency measurement in outpatient settings (Johansson et al., 2019). Various video detection methods exist in practice, which include marker-based (physical markers or sensors attached to the body) and marker-free methods (without relying on external sensors) (Ulate-Campos et al., 2016). Upon reviewing validation studies that qualified as phase 2/3/4, techniques like use of colored pajamas to facilitate limb movement tracking (Lu et al., 2013), identifying seizure sounds (Arends et al., 2016), muscle activity (Conradsen et al., 2012; Szabó et al., 2015; Milošević et al., 2016), periodicity in the luminance signal (Pisani et al., 2014; Cattani et al., 2017), and optical flow motion tracking (Karayiannis et al., 2005, 2006; Geertsema et al., 2018) have been reported. Vision-based motion recognition has been widely studied as the significance lies in its performance and robustness which is a critical functionality for decision support systems, particularly in clinical settings when diagnosing and managing epilepsy (Pediaditis et al., 2012). Literature (does not include neonates as outside the scope/intent-of-use of study’s algorithm) reports the overall sensitivity of video detection systems varying from 75 to 100%, positive predictive value over 85%, and specificity between 53–93% (Cuppens et al., 2012; Kalitzin et al., 2012; Pediaditis et al., 2012; Geertsema et al., 2018; van Westrhenen et al., 2020; Armand Larsen et al., 2022).

The application of artificial intelligence (AI) has significantly transformed the landscape of epilepsy phenotyping research, offering novel opportunities for automated and semi-automated analysis of various data modalities, with significant data reduction and the promise of clinical adoption of automated seizure detection and classification. The application of AI in clinical settings has shown tremendous potential in epilepsy diagnosis (Ahmedt-Aristizabal et al., 2023; Knight et al., 2024). AI-enhanced diagnostic methods may be trained to recognize cerebral localization from complex semiologic features, such as those observed in hyperkinetic seizures, which may not be reliably identified (or agreed upon) by clinicians (Ahmedt-Aristizabal et al., 2023). Despite the promise, challenges persist. The integration of AI algorithms into clinical practice necessitates robust validation, considering challenges such as dataset scarcity, natural clinical setting complexities, and the intricate nature of epilepsy semiologies (Ahmedt-Aristizabal et al., 2023; Karácsony et al., 2023). While vision-based motion analyses have demonstrated success in controlled environments, their reliability diminishes in noisy settings like epilepsy monitoring units (EMUs) and intensive care units. Factors such as varying lighting conditions, environmental occlusions (e.g., bed blankets, head wrapping), and interference from non-subject entities (e.g., clinicians, nurses) pose unique challenges (Ahmedt-Aristizabal et al., 2023; Karácsony et al., 2023). Deep learning models, though promising (Garção et al., 2023), are still in the early stages, struggling to recognize subject-specific semiologic categories and achieve fine-grained semiology recognition, crucial for distinguishing the stepwise progression of clinical features. These challenges extend to action recognition, where complexities in defining body part motions and variations between subjects hinder accurate automated detection. Moreover, some approaches that directly operate on RGB videos, exist with a possibility of privacy leakage of the sensitive patient data from videos, and the unrealistic wait for completion of the full seizure video to make predictions (Mehta et al., 2023). Other visual data modalities, including skeleton, depth, infrared, point cloud, and event stream, have their share of benefits and disadvantages as well (Sun et al., 2022). Within this study, we also took the opportunity to explore the challenges associated with 3D motion capture, including clinical personnel and soft occlusions such as blankets and adverse lighting conditions.

Per the International League Against Epilepsy (ILAE) and International Federation of Clinical Neurophysiology (IFCN) guidelines, use of clinically-validated wearable devices is recommended for the detection of generalized tonic–clonic seizures and safety indications (Beniczky et al., 2021). The guidelines emphasized the need to develop and validate automated detection systems for other seizure types and indications beyond patient safety. However, of the few devices that have ascertained their performance validation in phase 3 studies, all require patient contact, and may incur minor discomfort. Moreover, only limited evidence is available for the detection of motor seizures other than tonic–clonic seizures (TCS). There is a clear need to provide proof of utility and accuracy of the seizure detection devices for a broader spectrum of seizures, including hypermotor and other motor seizures (Beniczky and Jeppesen, 2019).

A novel contactless, marker-free, automated, video-based seizure detection system (Nelli) has been developed to aid clinicians in the detection of seizure events through a selection of relevant epochs based on biomarkers derived from audio and video (media) signals (Peltola et al., 2023). In a prospective, blinded, phase 3 study, wherein we had evaluated a solution based on an earlier version of the algorithm with a predefined detection threshold yielded a performance output of 93.7% sensitivity (95% confidence interval (CI): 69.8–99.8%) for the major motor seizures recorded, with a false detection rate (FDR) of 0.16 per hour (Armand Larsen et al., 2022). Of these seizures, 100% of the TCS and 80% of the hypermotor seizures were detected.

This study continues the previous phase 3 work with an improved motor seizure detection algorithm based on an ensemble of machine learning models trained on seizures recorded with Nelli in a home setting. Unlike the original study, which only focused on nocturnal periods, all recorded time periods where the patient was present in the scene (including day-time at-rest intervals) were included in the analysis. In addition to the generic statistical model used in the original study, a set of type-specific models contributed to the final detection score. The goal of our study is to assess the receiver operating characteristics of these new algorithmic models with major motor seizures created by choosing and grouping common ILAE types based on clinical use case and urgency. The performance is evaluated both for the set of predefined clinical seizure types, as well as all seizures of interest treated as a single major motor seizure group. Model stability within the dataset was assessed through cross-validation at the optimal threshold observed. We propose two use-case scenarios of the automated seizure detection system for clinical application: (1) Patient-safety: automated, real-time monitoring of videos in institutions; (2) Diagnostics: data-reduction of diagnostic home-video-monitoring, where epochs selected by the algorithm are reviewed by human experts, instead of reviewing the entire recording. We also note areas of future work, such as accessing the explainability and uncertainty of the model ensemble (and the models respective signals) when applied to a larger dataset.

While accurate differentiation between epileptic seizures and psychogenic non-epileptic seizures (PNES), can be challenging based on history alone (Naganur et al., 2019), the detection performance of these events was also included in this analysis. While subtle motor seizures can be detected by Nelli, a previous evaluation study indicated lower classification performance by hybrid (algorithm-human) review due to higher overall false detection rates (Peltola et al., 2023). Therefore, subtle seizure types such as single myoclonic jerks, epileptic spasms, and other very short seizures were out of scope for the present study and therefore excluded from the performance analysis.

## 2 Materials and methods

### 2.1 Study design

Study subjects were prospectively recruited patients referred to long-term VEM, as part of their diagnostic work-up, at two sites in Denmark: the Danish Epilepsy Centre, Filadelfia and Aarhus University Hospital, between June 2019 and July 2021. The study was granted approval by The Scientific Ethics Committee for the Zealand Region (SJ-756) on April 30 2019. All methods were performed in accordance with the relevant guidelines and regulations. Written informed consent was obtained from the patients or their parents/guardians (in case of children) prior to the study. Seizure labels were provided by the gold standard VEM methodology using a panel of three independent reviewers and blinded to the automated detection by Nelli. There was no restriction with the use of blankets by the subjects in the EMUs (Epilepsy Monitoring Unit). Use of wireless EEG also provided free movement of the subjects as they were allowed to leave the bed (as well as the video scene). Each seizure was labeled according to the ILAE 2017 seizure classification (Beniczky et al., 2017; Fisher et al., 2017), and seizures occurring outside of the recording area were excluded from analysis. The goal of combining different ILAE seizure types into distinct categories was to create groups of seizures that share a common clinical use case and care urgency. Five epileptic motor seizure groups were identified. PNES with a prominent motor component formed the sixth group, and was diagnosed according to the recommendations of the ILAE (LaFrance et al., 2013). These groups were clinically relevant since they have direct implications for decisions on patient management, and a measurable impact on the patient’s quality of life, such as causing disruptions to the sleep cycle. Some of these types may also lead to a focal-to-bilateral TCS.

Inclusion of seizure events was based on the following criteria:

1. The seizure type contained a motor component
2. The behavioral component of the event lasted for more than 10 s (cut-off selected as per the literature (Meritam Larsen et al., 2023) documented threshold for clinically relevant ictal phenomena, as well as widely accepted for electrographic seizures, suggesting clinical relevance for a video-based detection system)

Using the proposed standards for testing and clinical validation of seizure detection devices that identified four key features and their respective study designs for distinguishing between study phases (Beniczky and Ryvlin, 2018), the study met or exceeded most requirements for an explorative phase 2 study (Table 1). Although the hyperkinetic seizure group did not meet the said requirements of subjects and seizures, it was included as it had close proximity to the recommendation. However, the PNES group was included for illustration purposes only as it did not have a significant number of subjects.

**Table 1:** Feature and design recommendation of a phase 2 study.

Feature	Design	Recommendation	Study
*Subjects*	Simulation/ healthy subjects	Excluded	The study enrolled patients with suspected epilepsy.
	Number of patients with seizures	≥10	Out of 230 enrolled patients, 103 patients had seizures, with 81 patients experiencing motor seizures lasting >10 s. Number of patients in the seizure groups were as follows: TCS = 15, Tonic = 13 Automatism = 18, Unclassified motor = 40, Hyperkinetic = 7, PNES = 2
	Number of seizures	≥15	Total number of seizures identified during monitoring was 1,114, of which 334 seizures were motor seizures lasting >10 s. Number of seizures within each seizure group were as follows: TCS = 21, Tonic = 46, Automatism = 45, Unclassified motor = 164, Hyperkinetic = 14, PNES = 44.
*Recordings*	Conventional methods (already existing)	Excluded	The study uses a dedicated seizure detection device, instead of a conventional method.
	Dedicated device	Compulsory	The study used a dedicated device for seizure detection- Nelli.
	Continuous	Optional	Recordings were continuous, including daytime seizures detection.
	Multicenter	Optional	The study was carried out at two EMU sites.
	Offline/retrospective	Allowed	Patients were prospectively recruited in the study. The recordings were analyzed retrospectively.
*Analysis & alarms*	Training & testing using the dataset	Allowed	The training set patients were not included in the test set.
	Predefined algorithm and cutoff values	Not required	The study assessed a predefined algorithm, but cutoff values (threshold) was not predefined.
	Real time	Not required	Analysis was not real time.
	Blinded	Not required	Logging of the seizure detection time points was done blinded to all other data. The experts providing VEM labels were blinded to all data from the device.
*Reference standard*	Video or video-EEG recordings	Compulsory	The study used VEM as the gold standard.
	Information from patient and caregivers	Excluded	The study did not rely on the patient and caregiver for information.

### 2.2 Device description and mechanism

**Figure 1:**
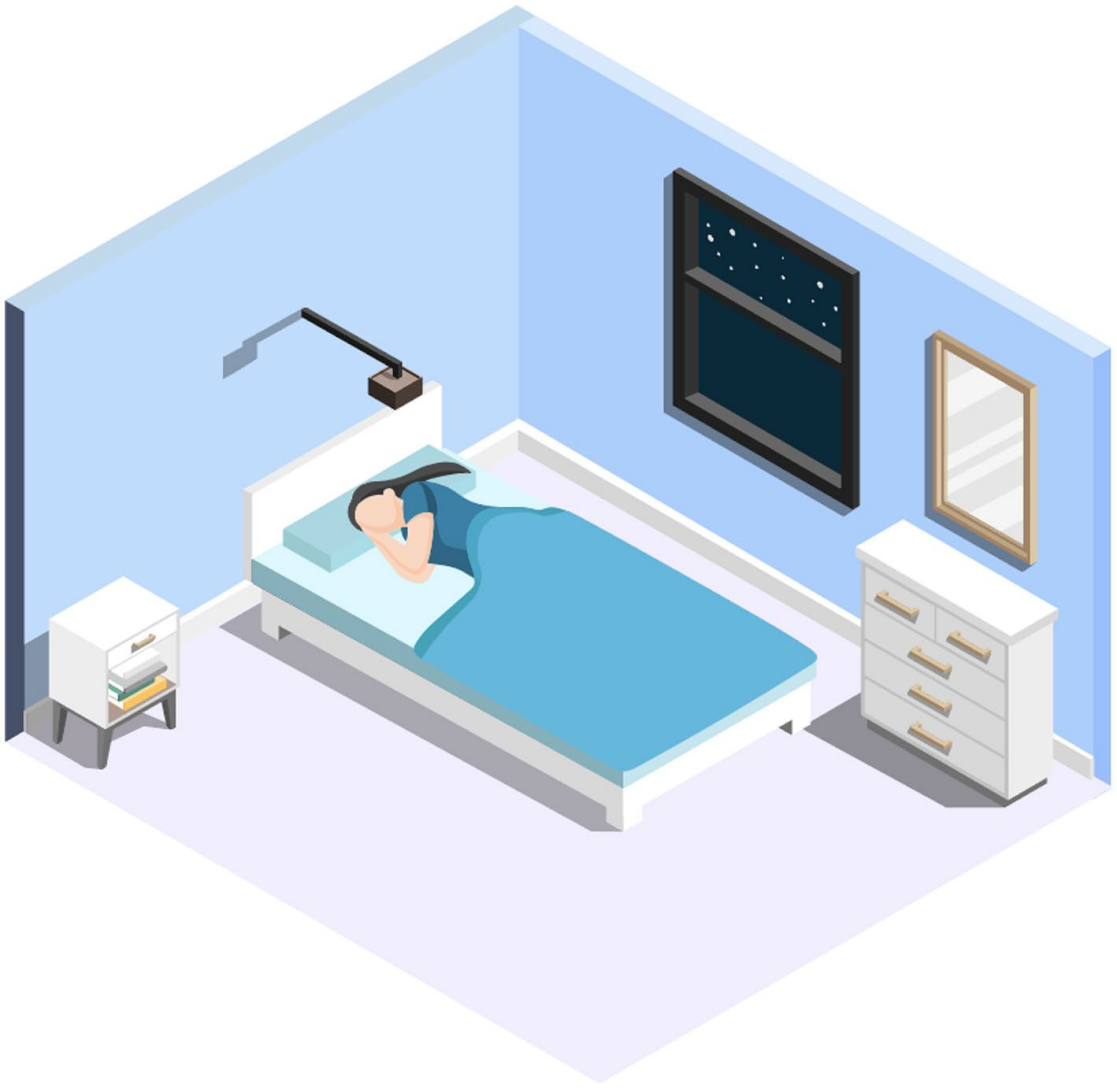
The Nelli recording device is shown mounted above a hospital bed.

A detailed description of the camera specifications can be found in an earlier publication (Ojanen et al., 2021). The automated seizure detection system (Nelli) consists of a stereo near-IR camera (Intel RealSense D435) attached to a compact industrial PC. As a silent and non-wearable device, it is designed to be less intrusive than other seizure monitoring technologies such as EEG, EMG, or wrist-worn devices. The raw data produced by the recording device is grayscale 30 frames-per-second (Hz) with low compression (VP9-encoded) stereo video at 1280×720 (“HD Ready”) resolution and accompanying compressed (Vorbis-encoded) 48 kHz stereo audio. Sound was captured using the built-in stereo microphone of an Intel NUC, a low-cost compact PC. The camera has field-of-view (FOV) of 87° × 58° for the stereo video sensors, allowing for capture of the complete bed area when the camera is mounted on the ceiling or wall above the bed. The use of the near-infrared spectrum allows it to capture clear grayscale images in the dark. The device has a global shutter, ensuring a fixed frame rate despite changes in lighting conditions. In the EMU environment, the camera was mounted in a fixed position 1.63 meters above the hospital bed (Figure 1). Unlike in other documented literature, the video was not cropped to a smaller bed area in this study. The computer’s software clips video events based on the presence of scene motion and transfers these clips to cloud storage for further processing. The system was not tested with other camera models, but may apply to hardware with similar characteristics given the design of the algorithm (described in section 2.4).

### 2.3 Test set

A total of 230 patients with suspected epilepsy were recruited to the study. Inclusion criteria were admission to long-term in-patient VEM in the EMU. Patients (Gavvala and Schuele, 2016) who did not have any motor seizures during the monitoring, and (Beghi et al., 2019) with completely failed recordings (device deficiency), were excluded from the analysis of sensitivity. All recruited patients and the entire monitoring time were used to determine the FDR.

Subjects’ ages ranged from 0 to 80, with a mean age of 23. The male-to-female ratio was 113:117 (51% female) (Table 2). The total number of events recorded by VEM was 1,114 among 103 subjects. Seizures lasting for more than 10 s were included in the study, as many short seizures are barely perceptible in video recordings (Peltola et al., 2023), implying 334 motor seizures reported from 81 subjects were within the scope of this analysis. This included 21 convulsive seizures, 14 hyperkinetic, 46 tonic, 45 automatisms, 164 unclassified motor seizures and 44 PNES. The events excluded were 218 non-motor events and 560 motor events lasting for 10 s or less. Table 3 summarizes the seizure type statistics as recorded by the gold standard seizure detection (VEM).

**Table 2:** Demographic characteristics of the patients.

Characteristic	*n* = 230
*Age range (years)*	
Infants (0–1)	4 (1.7%)
Children (2–11)	60 (26%)
Adolescents (12–21)	62 (26.9%)
Adults (22–80)	104 (45.2%)
*Mean age in years at consent (SD)*	23.2 (16.9)
*Gender*	
Male	113 (49.1%)
Female	117 (50.9%)
*Total events (subjects)*	1,114 (103)
*Total motor events > 10 s duration (subjects)*	334 (81)

**Table 3:** Clinical characteristics as seizure group summary of the patients (n = 81).

	Events	Subjects	Median age	Min age	Max age	Children (0–11)	Adolescents (12–21)	Adults (22 and above)	Seizure distribution (number of patients)		
									1 seizure	2 seizures	>2 seizures
***Convulsive (tonic–clonic) seizures***											
*I.D.01 (Focal to bilateral)*	20	14	31	14	72	0	3	11	10	3	1
*II.A.09 (Generalized)*	1	1	4	–	–	1	0	0	1	0	0
*Total*	21	15	26	4	72	1	3	11	11	3	1
*Duration in secs (min-max)*	59–1,057										
***Hyperkinetic seizures***											
*I.C.08 (Focal)*	12	6	37.5	5	50	3	0	3	2	2	2
*I.B.07 (Focal with impaired awareness)*	2	1	18	–	–	0	1	0	0	1	0
*Total*	14	7	26	5	50	3	1	3	2	3	2
*Duration in secs (min-max)*	15–424										
***Tonic seizures***											
*II.A.07 (Generalized)*	29	7	4	4	23	4	2	1	2	2	3
*I.C.05 (Focal)*	17	8	12	4	17	4	4	0	5	0	3
*Total*	46	13^*^	5	4	23	6^*^	6	1	6^*^	2	5^*^
*Duration in secs (min-max)*	11–947										
***Automatism seizures***											
*I.C.07 (Focal)*	43	17	26	11	72	1	2	14	8	2	7
*I.B.06 (Focal with impaired awareness)*	2	1	31	–	–	0	0	1	0	1	0
*Total*	45	18	26	11	72	1	2	15	8	3	7
*Duration in secs (min-max)*	13–298										
***Unclassified motor seizures***											
*I.C.01 (Focal)*	93	20	15	0	47	7	5	8	6	3	11
*II.A.01 (Generalized)*	36	7	26	12	46	0	3	4	2	3	2
*III.A.03 (Unknown onset)*	10	7	5	2	46	3	2	2	5	1	1
*I.A.01 (Focal aware)*	17	5	27	15	29	0	2	3	3	1	1
*I.B.01 (Focal with impaired awareness)*	8	3	45	27	58	0	0	3	1	0	2
*Total*	164	39^*^	23.5	0	58	10	10*	19*	15	8	17
*Duration in secs (min-max)*	11–2,292										
PNES^**^	44	2	34	34	40	0	0	2	1	0	1
*Duration in secs (min-max)*	18–148										

To aid in a compact representation of these various seizure types, the suggested codes (Beniczky et al., 2017) have been used.

^*^Total (n) is not equal to the group total as it excludes the duplicate subjects within the seizure group. ^**^The number of subjects within this group did not meet the recommended standard for seizure detection studies proposed in the current issue (Beniczky and Ryvlin, 2018) for a phase 2 study, but the group was kept for illustration purposes.

### 2.4 Seizure detection algorithm

Nelli’s seizure detection algorithm is based on a set of biosignals derived from physiologically-inspired video and audio analysis methods (Ojanen et al., 2021; Armand Larsen et al., 2022). Recordings used in training were collected from in-home studies using the same camera and microphone applied in the clinical investigation, with a total of 36 subjects and 2,570 expert-labeled motor seizure events (3,624 total seizure events). No samples from the test set were used in training or tuning the models. There were 12 pediatric subjects in this training set. Table 4 describes the demographic characteristics of the training set.

**Table 4:** Demographic characteristics of the training set.

Model	Subject count	Male:Female ratio	Age range	Mean age	Seizure events	Non-seizure events
Clonic	21	10:11	16–40	26.4	564	7,222
Hyperkinetic	9	5:4	16–46	31.0	490	4,353
Motor	31	17:14	17–61	28.6	2,570	10,949

In order to evaluate on a per-event basis (as opposed to, e.g., a time window basis), videos were temporally segmented to events based on zero crossing of a signal representing the depth-weighted motion content of the scene. The motion threshold was experimentally chosen based on the minimal perceptible movement above breathing, by observing samples from the training dataset. The events were recorded considering standard events such as clinicians or family members visible in the videos. Base event detections could arise from any movement in the scene, even if it does not originate directly from the study subject.

**Figure 2:**
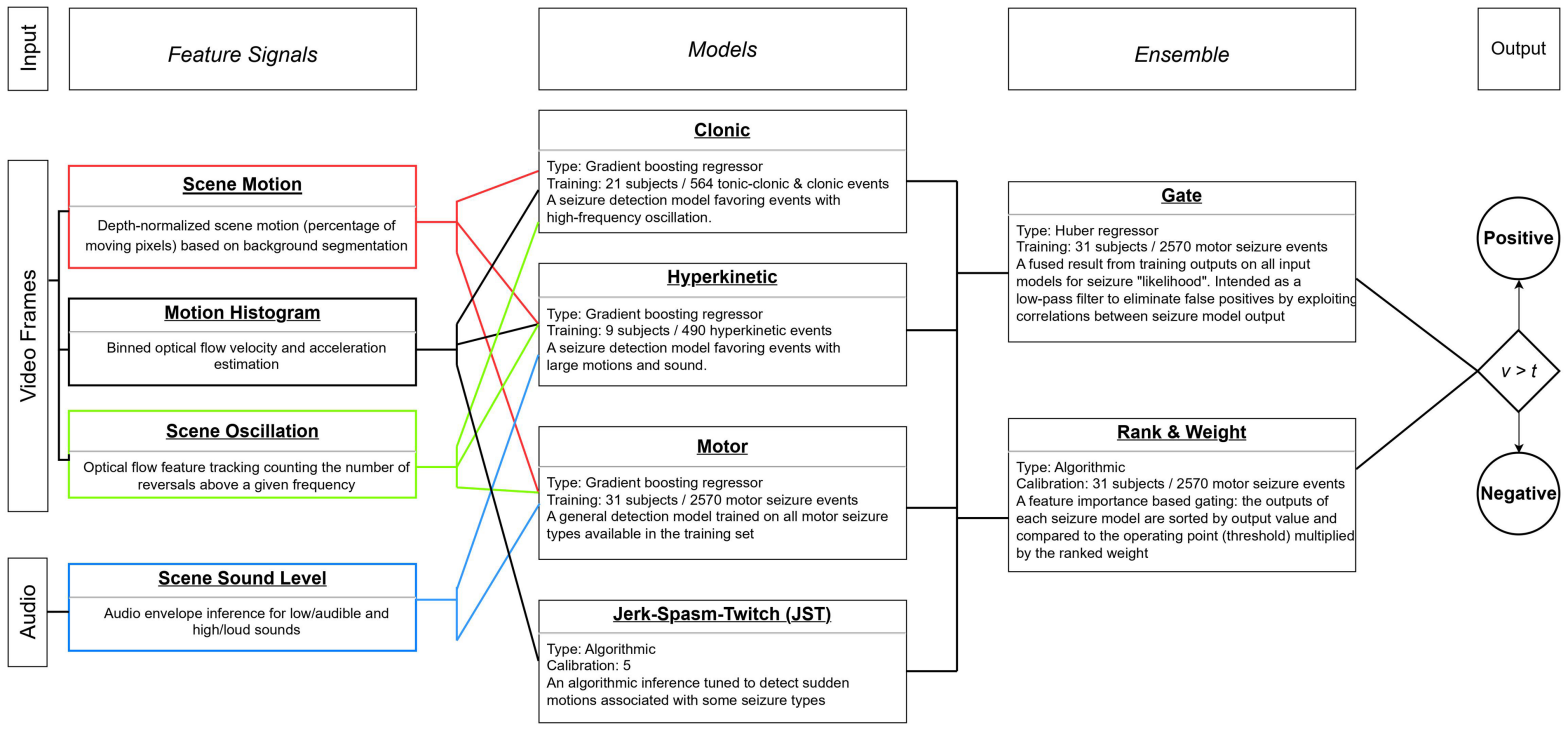
Diagram of feature signals, type-specific models, and final ensemble compared to operating threshold.

In addition to the depth-normalized motion information used for event segmentation, a mixed bag of additional signals was extracted from each event. The majority of signals are based on pixel change statistics (such as those derived from optical flow); as such, they are sensitive to sudden changes in lighting, shaking of the camera, motion from other people in the scene. Naturally, a multitude of choices in signal extraction methods, their configurations, dimensionality, sliding window length, region of interest, etc. all contribute to the quality of the signal and its ability to abstract a reliable biomarker for seizure detection. A full discussion of these signals is out of the scope of this study, but is based on the methods described in a previous published study (Ojanen et al., 2021). The sound level-based signals provide a good discrimination power for the motor model, the oscillation-based signals are exceptionally useful for the motor, hyperkinetic and clonic models, while the velocity and acceleration-based signals have high positive impact on the performance of the hyperkinetic model. Because patients present with oscillating limbs during clonic seizures and the clonic model inputs optical flow-based motion signals, this model prefers motions with high-frequency oscillations during the seizure events as stated in Figure 2.

The signals were fed through an overlapping 20-s sliding window with 50% overlap into an ensemble of algorithmic and machine learning models, each with its own feature engineering and training dataset. Other sliding window lengths and overlaps were experimentally explored during model design, with the chosen parameters based on observations of typical behavioral duration of seizure activity and a desire to keep the potential maximum latency of the system low. The models were trained separately before creating the model ensemble, and the training dataset for every model was a subset of the in-home patients exhibiting the relevant seizure type of the model. For example, the clonic model was built by positive and negative samples chosen from the in-home recordings of patients with annotated clonic seizures. A model outputs a probability of seizure value (0 to 1) for every sliding window of an event and the event score is the maximum of these probabilities. In order to be considered a positive sample, all models in the ensemble must pass their ranked threshold. Then these scores are fused together by a weighted gating of all model’s event scores, calculated from single-valued feature importances against the training set, to arrive at the final “seizure likelihood” score. Notably, this means that the base motion segmentation event serves as an aggregated time range for multiple model predictions, each limited to a 20-s window, and limited to the extracted signals during that time range. Therefore, the ensemble is not aware of the entire content of the event, but relies on the maximum value output for the collection of sliding windows.

Accordingly, although four models are trained on specific seizure types, the system does not output distinct probability values for each seizure type, but rather a single seizure probability for each event. This probability value can then be used at different thresholds depending on the use case and target seizure types. The determination of these optimal threshold values, dependent on the seizure group under study, is explored in the following section. The series of extracted features, the participating models in the ensemble with their training characteristics, and a description of the final gating process have been described in a flowchart diagram presented in Figure 2.

### 2.5 Performance analysis

To assess the system’s performance, seizure labels were compared to the Nelli event detections by intersection of timestamps. A hit or true positive (TP) event identified by the system was defined as a detection that intersected with the VEM label. A false positive (FP) event was defined as an event identified by the system that did not intersect with a VEM label. A false negative (FN) event was defined as a positive VEM label that was not identified by the system. TP and FP events were identified independently for all seizure groups. Sensitivity was calculated by dividing the number of TP events and total VEM positive events for the group, while FDR was calculated as FP per hour of recording. The effectiveness of different video detection systems were compared (Ulate-Campos et al., 2016) and a single acceptable performance was adopted for the study. The individual performances of the seizure groups were considered satisfactory if the sensitivity was equal to or exceeded 70% and individual FDR was equal to or below 7 per hour. A comparative analysis of all the seizure groups was also carried out to determine combined optimal thresholds of the algorithmic model. False alarm rate was also reported, which was calculated by dividing the number of FP events and total VEM negative events for each seizure group.

The performance of the algorithmic model was presented using sensitivity (95% exact binomial CI) and FDR (95% bootstrapped CI) parameters. Interpolation of the CIs for sensitivity was done using Piecewise Cubic Hermite Interpolating Polynomial (PCHIP) interpolation for attaining smoother plots. Geometric mean FDR (subject-level) and 95% CI were reported for seizure groups with skewed data, at lower thresholds. Overall FDR (event-level) was used at threshold equal or higher than 0.85. Due to high precision at higher thresholds, no FP events were reported for few patients and the corresponding geometric mean could not be computed. Thus, the use of overall FDR at those thresholds was advisable. An individual FDR for each seizure group was also reported which included events from the patients within the group only and seizures outside the group were treated as FP. As an additional outcome measure endpoint, detection latency was also calculated, which is defined as the difference (in seconds) between the model threshold time and seizure onset time as determined by vEEG. It was summarized using non-parametric descriptive statistics.

Patient and screen occlusion were also explored in the study. Different occlusion scenarios were established and event distribution were reported. Performance of each occlusion scenario were reported and associations were explored through statistical testing.

A series of k-fold cross-validations was performed to explore the stability of the model when subsampled. The resulting median performance and interquartile ranges were compared between the subsamples, providing some descriptive statistics of the model’s performance variability within the population and hinting at its potential generalizability for an unseen dataset.

Data analysis and visualization were carried out using Python (version 3.10.6) with pandas, matplotlib, numpy, seaborn, sklearn and scipy packages.

## 3 Results

### 3.1 Absolute performance of seizure groups

**Figure 3:**
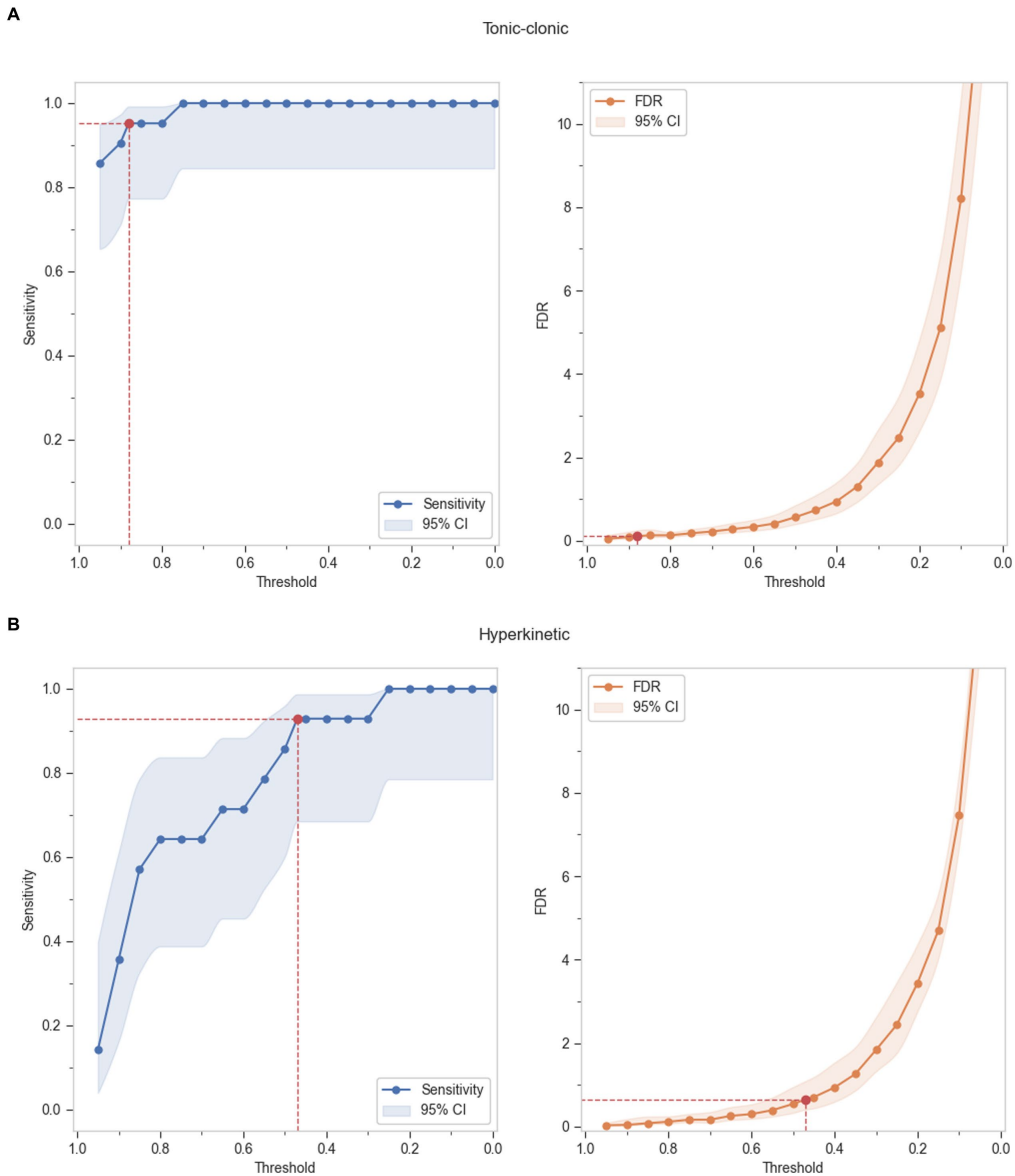

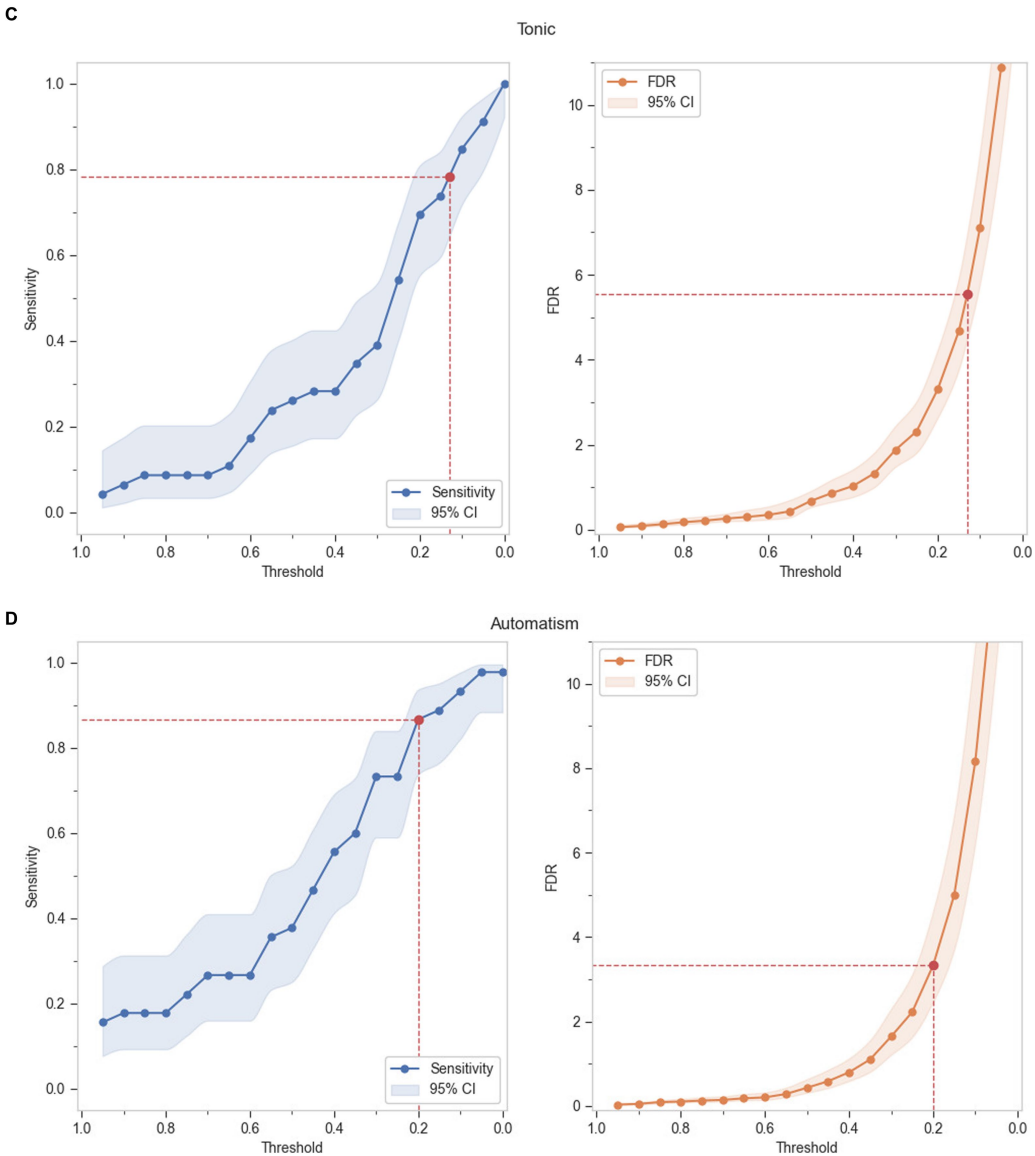

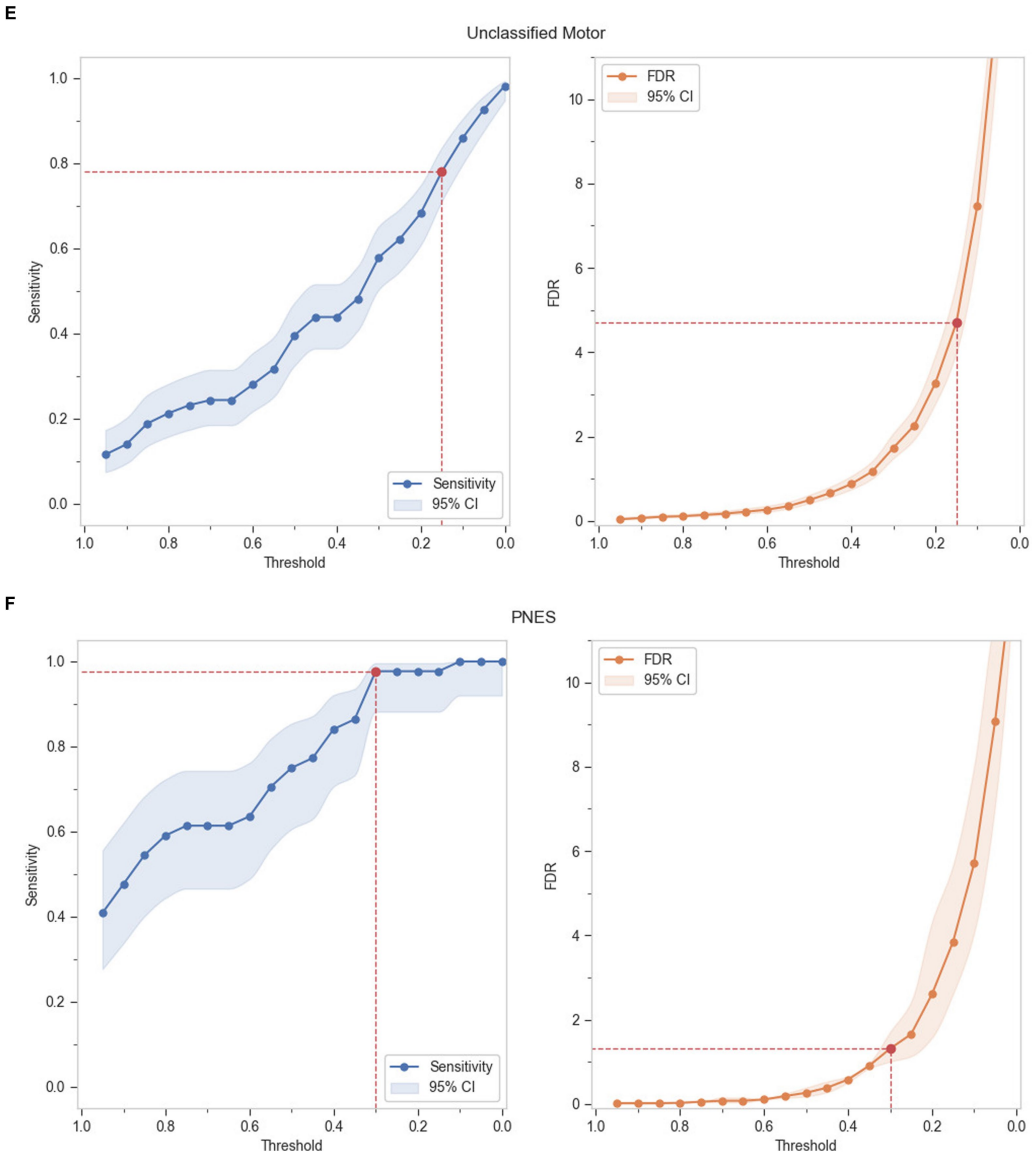
Absolute performance of the seizure groups at incremental thresholds - **(A)** Tonic–clonic seizures, **(B)** Hyperkinetic seizures, **(C)** Tonic seizures, **(D)** Automatisms, **(E)** Motor seizures, **(F)** PNES.

Grouped TP and FP events by ILAE seizure types, as defined in Table 3, was used to calculate sensitivity and FDR per hour for each seizure group. A range of thresholds was evaluated at suitable increments comparing sensitivity and FDR per hour. Figure 3 shows the absolute performance of the seizure groups in terms of sensitivity and FDR per hour respectively, against detection thresholds.

The optimal thresholds were determined from the individual seizure group’s performance output, which best balanced the sensitivity and specificity, and yielded the maximum sensitivity for each group while detecting a lower FDR than 7. Upon comparison with the VEM labeling, sensitivity was found to be higher than 70% for all the seizure groups (Table 5). The model performed best for convulsive and hyperkinetic seizures, where only a single seizure event was missed. Tonic, automatism, and unclassified motor seizure groups also had an above-satisfactory performance at lower thresholds, while only five events were missed in the PNES group (note that only two subjects were present in this group, and therefore are not likely representative of this seizure type). The population FDR was as low as 0.09 and 0.64 per hour for TCS and hyperkinetic seizures, while the highest detection was recorded in the tonic seizures as 5.87/h. False alarm rate was reported as low as 0.003 in the TCS group, with a maximum rate of 0.26 in the tonic group.

**Table 5:** Nelli performance summary of seizure events analyzed.

Seizure group	Subjects with events	Optimal threshold	VEM positive events	Nelli true positive detections	Sensitivity (95% CI)	Group FDR/h (Bootstrapped 95% CI)^a^	Population FDR/h (Bootstrapped 95% CI)^b^	Population False Alarm Rate	Detection latency in secs (median [Q1, Q3])
Convulsive	15	0.88	21	20	95.2% (82.4, 100%)	0.11 (0.06, 0.18)	0.090 (0.077, 0.103)	0.003	−3.3 [−19.5, 0]
Hyperkinetic	7	0.47	14	13	92.9% (68.5, 98.7%)	0.65 (0.39, 1.07)^*^	0.64 (0.59, 0.69)^*^	0.023	−14.3 [−16, −4.3]
Tonic	13	0.13	46	36	78.3% (64.4, 87.7%)	5.54 (4.45, 6.91)^*^	5.87 (5.51, 6.23)^*^	0.257	−4.8 [−21.8, 0.1]
Automatisms	18	0.2	45	39	86.7% (73.5, 97.7%)	3.34 (2.44, 4.57)^*^	3.34 (3.12, 3.58)^*^	0.134	−12.0 [−28.0, 0.2]
Unclassified motor	39	0.15	164	128	78.0% (65.4, 90.4%)	4.64 (3.88, 5.54)^*^	4.81 (4.50, 5.14)^*^	0.205	−2.3 [−17.4, 1.7]
PNES	2	0.3	44	43	97.7% (97.7, 100%)	1.31 (1.01, 1.71)^*^	1.73 (1.61, 1.86)^*^	0.065	−3.3 [−9.2, 0]

a Includes events from the patients within the seizure group; seizures outside the group are treated as FP.

b Includes events from the entire population (n = 230); seizures outside the group are not treated as FP.

^*^Geometric mean and 95% CI.

The median detection latency for TCS, tonic seizures, unclassified motor seizures and PNES were well-aligned to the vEEG-labeled time, and were over −10 s for hyperkinetic seizures and automatisms. This shows that the optimal threshold tuning for the seizure groups activated the moment when the motor component of the seizure became more prominent than normal sleep movement or all seizures, with the possibility of getting triggered by more common movement events in case of hyperkinetic seizures and automatisms.

The prioritization of convulsive seizures as the primary focus necessitated an examination of potential oversights within this seizure group. It was observed that the one non-TP seizure event (1/21) in the convulsive seizure group, while not entirely missed, was identified as a medium-priority seizure event (characterized by hyperkinetic, tonic, automatisms, and unclassified motor manifestations) at the designated threshold. In terms of its implications for the treatment trajectory (assuming Nelli would be the sole seizure monitoring device), the deduction drawn herein suggests that the patient experiencing this missed convulsive seizure might have faced a potential delay in treatment, albeit without any subsequent alteration in the ultimate clinical outcome.

### 3.2 Combined performance of seizure groups

**Figure 4:**
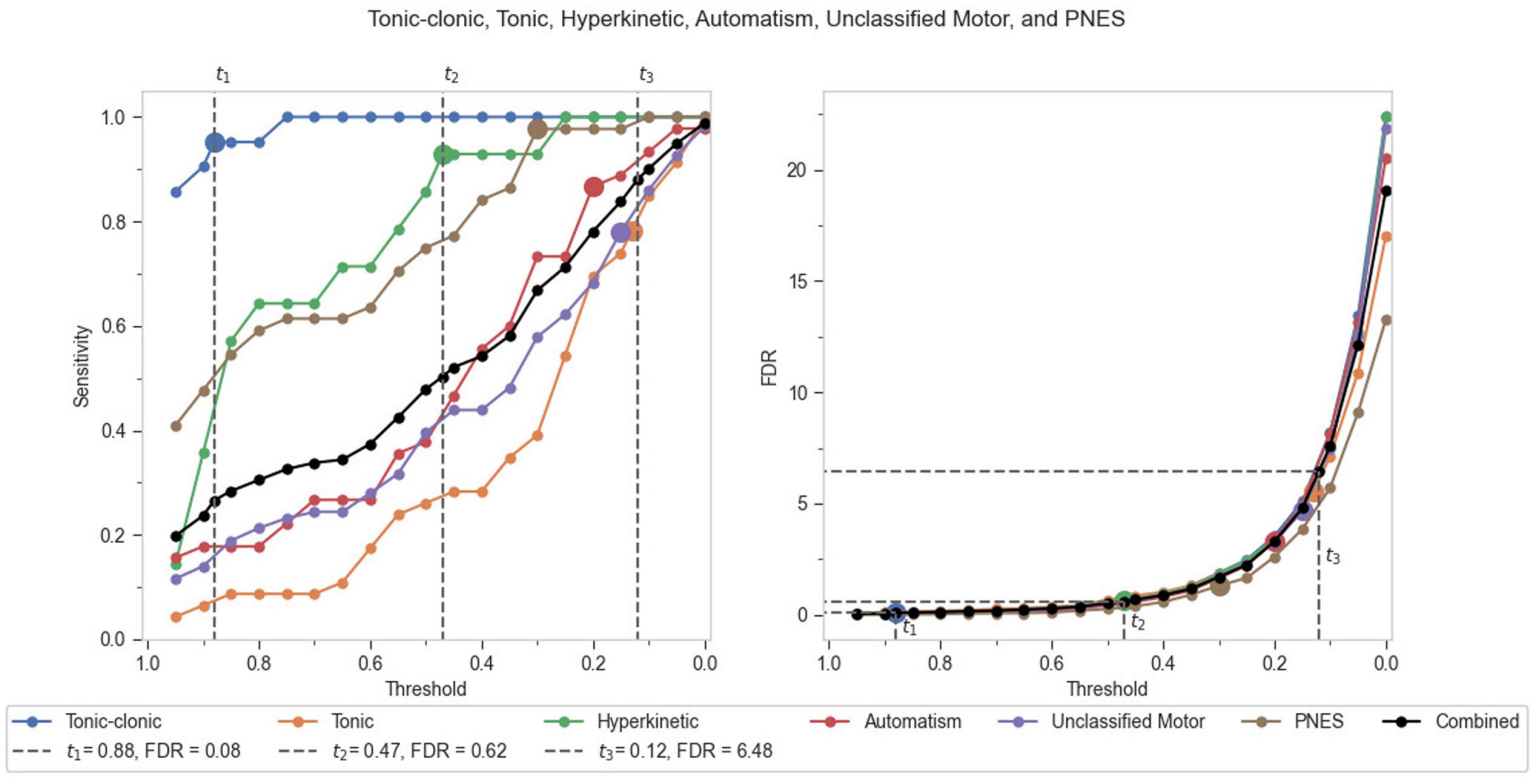
Comparative performance of the seizure groups in terms of sensitivity and FDR per hour against incremental thresholds.

After an absolute performance analysis of the individual seizure groups, a comparative analysis was also conducted (Figure 4). A combined performance output of all seizure groups is presented as a black line plot for sensitivity and FDR per hour in Figure 4. This combined plot for FDR was found to follow the trend of individual seizure group absolute performance, and thus served as the best representation for deriving recommended thresholds for the algorithmic model as a whole. Three recommended thresholds were derived from the comparative analysis as *t_1_* (0.88)*_,_ t_2_* (0.47)*, and t_3_* (0.12). The first recommended threshold, *t_1_*, would be useful in detecting most of the convulsive (TCS) seizures (95.2% sensitivity), with an FDR that is likely acceptable in an urgent care facility (0.09 per hour). The second recommended threshold *t_2_* would perform best in screening TCS along with hyperkinetic seizures (92.9% sensitivity) in patients, with a comparatively higher but acceptable FDR in an EMU setting due to the presence of monitoring staff (0.62 per hour). At this threshold, the algorithm can be seen yielding 100% sensitivity for TCS, which is far below the *t_1_* threshold. This denotes that *t_2_* can be deemed a reasonable place to maximize patient safety with respect to convulsive seizures, coupled with the detection of maximum hyperkinetic seizures. The third recommended threshold *t_3_* would work well in the detection of all major motor seizures under investigation (88% sensitivity), while keeping the FDR below 7 (6.48 per hour).

### 3.3 Occlusion scenarios and their impact

The different sources of patient occlusion and scene disturbance include the use of a blanket, other people, and disruptive lighting changes. Table 6 provides a detailed description of the occlusion scenarios identified within the dataset, with the aim of assessing if there is a significant association between the types of disturbance and sensitivity reported in each scenario.

**Table 6:** Different patient and scene occlusion scenarios observed during labeled seizures.

Scenario	Description
1	No occlusion
2	Blanket (covering any part or limb, for entire video or removed later)
3	Person between patient and camera within 10 s of seizure onset (occluding at least 10% of the patient as judged visually)
4	Person between patient and camera within 20 s of seizure onset (occluding at least 10% of the patient as judged visually)
5	Person between patient and camera within 20 s of seizure onset (occluding at least 10% of the patient as judged visually)
6	Another person in the scene at any time during the seizure
7	Disruptive lighting changes (e.g., the room lighting is changed suddenly, or a moving external light source is introduced in the scene)

Events with multiple sources of occlusions were also recorded. A total of 272 events (of 334) were recorded with blanket occlusion, of which 129 events had only occlusion by blanket. Similarly 61 (of 334) events were recorded with external light source interruption in them, of which 2 were exclusive. Events that involved another person occluding the patient numbered 193 (of 334), of which 40 were exclusive of other disturbances. The scenario-wise sensitivities were recorded for both overall groups and their respective exclusive groups (Table 7). Association between the scenario type and their respective sensitivities (at the optimal generic threshold for all motor seizure types) were analyzed using the chi-square test for variable independence. Under the conditions with only blanket occlusion, the model detected 76.7% of the seizures, while 95% of the seizures were detected by the model when the event reports occlusion through another person. In conditions with overlapping disturbance types (blanket, another person, disruptive lighting), the model detected 96.3% events (*p* < 0.001). As none of the exclusive scenarios reached statistical significance for variable independence, when considered individually, the data does not support the conclusion that any of the observed disturbances exert a significant challenge to the overall study sensitivity or the functionality of the algorithm.

**Table 7:** Performance recorded in different occlusion scenarios (presented both inclusive and exclusive of overlapping disturbance types).

Scenario	Inclusive TP/T	Sensitivity	Exclusive TP/T	ME Sensitivity
1	7/8	87.5%	–	–
2	237/272	87.1%	99/129	76.7%
3	47/48	97.9%^*^	13/13	100%
4	14/14	100%	2/2	100%
5	77/77	100%^***^	12/12	100%
6	48/54	88.8%	11/13	84.6%
3/4/5/6	186/193	96.3%^***^	38/40	95%
7	60/61	98.3%^**^	1/2	50%

TP, True Positive, T, True events as recorded by video-EEG monitoring Chi-square test for independence was used to check the association between the scenario and the detection by model. Corresponding *p*-values have been shown as ^*^p < 0.1, ^**^p < 0.01, and ^***^p < 0.001.

While it is expected that scene disturbances may have an adverse effect on the performance of the algorithm, the correlation of these to the algorithm’s sensitivity in this dataset is inconclusive. A closer analysis of the signal quality of the derived biomarkers in selected samples would be warranted in order to better understand the effect of these disturbances and how to mitigate the addition of noise to the underlying physiological biomarkers. It would also be warranted to observe the sensitivity effect over a range of operating points, as the association may be more prominent as the threshold is raised. Furthermore, the strong statistical association with increased sensitivity in some types of disturbances may be attributed to additional signal content, or may simply be correlated to the underlying clinical explanations, such as larger (and therefore easier to detect) seizures leading to faster intervention from hospital staff.

### 3.4 Model stability

The 10-fold, 5-fold and 3-fold cross-validation (CV) strategies were used to test the model stability at the observed optimal threshold (0.12). The study population included patients with seizures of interest (n = 81) for sensitivity and all enrolled subjects (n = 230) for FDR. The cross-validation of the study test set utilized patient-level splits, and it was split into k consecutive folds of “In-sample (IS)” and “Leave-out-of-sample (LOOS)” sets and a median performance assessment was reported for each cross-validation strategy, along with first quartile and third quartile as a measure of variability. 10-, 5- and 3-fold CV divided the study population into two subsets of 9–1 folds, 4–1 folds, and 2–1 folds, for IS and LOOS sets, respectively, with 10, 5 and 3 iterations. Event-level sensitivity and FDR were calculated for each iteration and a statistical average (median sensitivity and FDR) along with a measure of variability (IQR) were reported for all k-fold strategies. The resulting number of patients in each set and the performance assessment of the IS and LOOS sets have been presented in Table 8. As each patient reported multiple types of seizures, class distribution was not a critical factor in patient-level cross validation.

**Table 8:** Model stability assessment through cross-validation and comparison of sub-sampled data variabilities.

CV strategy	Number of subjects per fold	Performance metric	IS set	LOOS set
*10-fold*	IS: 73 LOOS: 8	Median Sensitivity (Q1, Q3)	87.7 (87.1, 88.5)	90.9 (82.0, 96.5)
	IS: 207 LOOS: 23	Median FDR (Q1, Q3)	7.46 (7.38, 7.47)	7.07 (6.86, 7.74)
*5-fold*	IS: 65 LOOS: 16	Median Sensitivity (Q1, Q3)	87.8 (87.6, 88.0)	87.9 (87.1, 88.7)
	IS: 184 LOOS: 46	Median FDR (Q1, Q3)	7.44 (7.44, 7.45)	7.33 (7.31, 7.36)
*3-fold*	IS: 54 LOOS: 27	Median Sensitivity (Q1, Q3)	87.8 (85.8, 89.5)	88.1 (84.3, 90.6)
	IS: 153 LOOS: 77	Median FDR (Q1, Q3)	7.44 (7.40, 7.45)	7.38 (7.37, 7.46)

The median sensitivity and FDR were similar across the folds in the IS set, despite the high variability within the data. At lower values of k, variability in the IS set remains low, suggesting that the model is generally stable for the study dataset and is not strongly affected by outliers. The results of the cross-validation indicate that the LOOS set exhibited performance metrics that were consistent with those observed in the IS set. This suggests that the model performance is stable across the assessed population, and might be expected to offer a similar level of performance on an unseen dataset.

## 4 Discussion

In this phase 2 study, the operating characteristics of the automated, video-based seizure detection algorithm of Nell were tested in an EMU setup against the gold standard (VEM). We found that different motor seizures across the epilepsy spectrum, as well as a selection of PNES, were detected by the system at a satisfactory performance level for manual video-based diagnostic review. Furthermore, for the detection of convulsive seizures, the FDR was sufficiently low for real-time application of the system as a seizure alarm. The detection latency of the model was well-aligned to the seizure onset time determined by the gold standard (under −15 s for all groups).

At optimal thresholds (balancing sensitivity versus FDR), the system detected tonic–clonic seizures, hyperkinetic seizures, tonic seizures, automatisms, unclassified motor seizures, and PNES with sensitivity higher than 70% and FDR lower than seven per hour. Using the comparative analysis, three recommended thresholds were sought for the combined performance of the seizure groups. These thresholds (*t_1_*, *t_2_*, *t_3_*) have been reported to accurately detect TCS, hyperkinetic seizures, and other motor seizures under study, respectively. When considering a single generic threshold, all major motor seizures were detected with 88% sensitivity, 6.48 FDR/h at 0.12 threshold. These results are indicative of the recommended pre-specified thresholds for the automated seizure detection system and the performance yield achievable from it. These results corroborate the performance yield achieved in the phase 3 study, wherein all 11 TCS (100% sensitivity; 95% CI: 71.5–100%) and four out of five (80% sensitivity; 95% CI: 28.4–99.5%) hypermotor seizures were detected among 51 patients (Armand Larsen et al., 2022). However, in the previous study that analyzed only nocturnal recordings, the FDR for all nocturnal motor seizures among the 181 total patients was reported as 0.16 per hour. The increase in FDR (*t_2_* threshold) in the present study results from the inclusion of daytime motor seizures at rest apart from the nocturnal seizures. Although the sensitivity was relatively similar, with 92.9% sensitivity among 35 seizures, compared to 93.7% sensitivity among 16 seizures in the phase 3 study. This level of performance is clinically relevant when considering different real-world use scenarios, including enhancement of a hybrid (algorithm-human) system for retrospective detection and classification of motor seizures (Peltola et al., 2023), as well as for real-time detection of TCS and hyperkinetic seizures in home, institutional care or EMU settings (Armand Larsen et al., 2022). It is notable that the model design may leave “performance on the table,” as it does not yet leverage uncertainty in statistics when applying model weights. When applied to a larger training dataset (such as the test set in this study), the ensemble may perform significantly better by leveraging knowledge of the types of signal profiles where the individual models have the highest levels of certainty. Such a study would also allow for examining the performance of individual models (and their underlying signals) in a systematic way to provide better explainability for the system.

The performance yields and detection latency observed in both convulsive and hyperkinetic seizure groups align closely with findings from previous studies investigating seizure detection through automated video analysis employing optical flow signal (Geertsema et al., 2018; van Westrhenen et al., 2020). Furthermore, the sensitivity demonstrated for convulsive seizures is coherent with the performance reported by wearable seizure detection devices validated in phase 3 studies (Beniczky et al., 2021). Beyond the detection of high-priority TCS, the algorithm successfully identified automatisms with a sensitivity exceeding 70%, similar to results reported in another study utilizing wearable sensors (Tang et al., 2021).

Screening and differential diagnosis are essential components in the detection of seizures and the correct implementation of treatment (Elger and Hoppe, 2018). One of the major obstacles in the classification and differential diagnosis of suspected epileptic seizures is the patient’s inability to accurately describe key features of these events (Mielke et al., 2020). Additionally, the ability to monitor seizure activity over time (Duun-Henriksen et al., 2020) also favors a better understanding of seizure types, frequency, and severity, helping clinicians to better understand the patient’s condition and to assess the effect of medical interventions (Basnyat et al., 2022).

In an urgent care setting, convulsive seizures are highly undesirable and accurate detection is imperative. In such a clinical case, the first threshold *t_1_* is recommended, as the corresponding FDR is likely acceptable in a real-time monitoring scenario. The second recommended threshold *t_2_* could potentially be used from real-time monitoring in cases where a higher FDR is acceptable (e.g., in an EMU with monitoring staff or in some residential care units) while detecting TCS along with hyperkinetic seizures among the patients. Hyperkinetic seizures often result in large-scale body movements, loss of consciousness, and can be confused for non-epileptic seizures due to the similarities in symptoms and therefore, are challenging to accurately differentiate (Lee and Khoshbin, 2008; Anne, 2013). Moreover, these seizures can lead to patient injury due to collapse and falling out of bed. The third recommended threshold *t_3_*, with a sensitivity of 88% and FDR of 6.48 per hour, is still relevant in detecting other concerned epileptic seizures and unclassified motor seizures, as well as PNES, taking into consideration the patients with developmental disorders, dissociative disorders, or intellectual disability tend to have higher FDRs due to more frequent or repetitive idiosyncratic movements. This threshold is especially relevant for the enhancement of a hybrid (algorithm-human) system for retrospective detection and classification of motor seizures (Peltola et al., 2023).

One recognizes the rapid advancements in deep learning methodologies in the context of video-based detection methods; however, it is imperative to acknowledge that despite these strides, there remains a notable gap in the clinical validation of these techniques, particularly on suitably large clinical datasets (Ahmedt-Aristizabal et al., 2023). Of the majority of seizure detection devices that are available, most have been developed using the same datasets for training and testing in the algorithm development phase, creating an inclusion bias. This limits the validity of the algorithm’s performance due to potential overfitting and may produce misleadingly high performances (Johansson et al., 2019; Ahmedt-Aristizabal et al., 2023). On the contrary, the results of our study stand valid as there was no overlap between the patients in the training and test datasets. A clear separation of patients between training and testing sets ensures that all recorded instances of a particular patient are exclusively assigned to either the training or testing set and is essential to accurately appraise the system’s ability to generalize (Ahmedt-Aristizabal et al., 2023). However, the cut-off thresholds were not predefined (but rather explored as part of the study design), and therefore may not necessarily generalize to another dataset. Achieving generalization to unseen subjects has consistently proven challenging in the realm of medical machine learning, primarily attributed to the substantial variability observed among subjects (Ahmedt-Aristizabal et al., 2023). Despite the absence of a blinded test set, some exploration into the model’s stability provides evidence of its generalizability: a cross-validation of the model’s outputs showed that it gave consistently similar results, even when highly subsampled. Despite the high variability of data, the average performance remains similar across folds, suggesting that the measured performance is not highly dependent on the dataset.

Seizure semiology is often prone to inter-observer discrepancy due to qualitative criteria reliance. A system capable of measuring the seizure features quantitatively would allow detection changes in seizure severity or seizure propagation. With Nelli, quantitative analysis of movements is applied to the media data, to develop objective summaries of semiological components of identified events. This helps in forming the backbone of a correct categorization of seizures (Wolf et al., 2020), which was not possible in the self-reported paradigm. Furthermore, the presence of a video recording of a seizure allows the clinician to review the seizure itself, which is not possible with non-video seizure detection systems (Amin et al., 2021). These features have practical implications in tasks such as presurgical workup and therapy outcome assessment.

Clinical validation becomes paramount to ensure the reliability and effectiveness of AI-based systems in real-world scenarios. Despite the impact of occlusions in the study, the model showed robust performance. While multi-camera systems offer a partial solution to occlusion challenges and potential enhancements in tracking performance, the practicality of clinical implementation requires considerations of cost efficiency and minimal spatial footprint. Clinical monitoring rooms, typically designed for maximum patient capacity, are congested with various clinical apparatuses, thereby imposing constraints on available space and camera mounting positions. These factors necessitate the restriction of the camera count, making the utilization of a single camera the prevailing solution for monitoring in clinical settings (Karácsony et al., 2023). The seizure onset detection latency achieved by the model was also in line with (Garção et al., 2023), that is between 5 and 35 s, although most markerless video-based methods do not specify latency. Another consideration is the inclusion of daytime seizures, showing the model’s capability of objectively recording seizure counts and characteristics holds immense value, not only for seizures occurring during sleep but also for those manifesting while the patient is awake (Ahmedt-Aristizabal et al., 2023). The study models do not operate on the video frames directly, reducing privacy issues when storing derived signals as opposed to video. While the model was not evaluated for real-time usage as it used discrete analysis, the use of the 20 s, 50% overlapping sliding windows for data processing would allow detection to occur without the unrealistic wait till the end of longer seizures (Mehta et al., 2023), should a continuous modeling system be adapted as an area of future improvement.

Nelli is non-obtrusive and intended to provide clinicians with video data as an adjunct for diagnostic categorization by the reviewing physician. While Nelli is not currently designed to operate as an alarm, the algorithms can potentially operate continuously due to the use of sliding windows. Therefore, future iterations of the product might feature real-time notifications for the detected events, provided that computational requirements are met for continuous inference. In an institutional setting, such as hospitals and residential care facilities, it may be possible to implement the real-time seizure alarm using Nelli. This could significantly reduce the need for long and continuous video surveillance during the night shift, as personnel would be present to act in the event of an alarm. Such a system could improve the efficiency of the care staff by reducing their workload.

Another interesting issue to note in the devices that detect TCS is the use of an oscillation measurement as a biomarker. This corresponds to the disadvantage that the seizure is first detected during its clonic phase, and this higher latency makes the system less impactful in an alarm, despite being highly specific. On the other hand, if a multimodal model like Nelli was to be integrated in an alarm system, the tonic biomarkers (sudden movement and sound) could potentially detect the seizure’s onset earlier.

The study also has several limitations. There is a possible gap in the age distribution among the patients included, with only 27% under the age of 11 (infants and children) included in the study. This section of patients was accountable for 79.7% of the short seizures in the study. A subgroup analysis was however not performed to avoid type I and II errors due to multiple hypothesis testing and inadequate power. Despite the high number of recruited patients, there was a relatively low number of patients and seizures under hyperkinetic and PNES groups. The authors identify the training coverage in terms of biomarker selection as a window of opportunity for further improvement, wherein a more comprehensive training set will help in training the algorithm even better. With respect to the device mechanism, using video for event detection restricts the area of interest, leading to challenging detection in case the patient leaves the scene. In such scenarios, the seizure recognition would completely be based on the sound signal. The illustration of variability of the model’s performance when subsampled was performed on the same, seen test set as there was no further “unseen” data to evaluate on, we certainly anticipate future phase 3 studies to evaluate this model (or a future revision of the model) on a new dataset.

The challenge to tackle is to improve the specificity of prominent seizures within the study by decreasing false detections, which would be one of the next steps for development. Furthermore, the dataset had many short seizures (176 motor seizures of interest lasting for less than 10 s) which were excluded given the inclusion criteria set for the study. Should the modeling be adjusted to accommodate these shorter seizures, they could be evaluated for their performance.

In conclusion, this study explores the performance of the AI-based analysis of audio-video recordings using the Nelli system with respect to different seizure types and at different operating points for monitoring motor seizures at rest. The findings of this study show that Nelli as a seizure monitoring system device can improve the correct detection of seizures as well as differentiate between seizures and non-seizure events through data-driven analysis. Our results suggest that the performance of the Nelli system is clinically applicable for use as a seizure screening solution in diagnostic workflows, for both real-time detection of convulsive seizures, and for improving the efficacy of a hybrid (algorithm-human) system for reviewing video recordings by significantly decreasing the workload for accurate classification of all motor seizures lasting longer than 10 s.

## Data availability statement

The raw data supporting the conclusions of this article will be made available by the authors, without undue reservation.

## Ethics statement

The studies involving humans were approved by The Scientific Ethics Committee for the Zealand Region (SJ-756). The studies were conducted in accordance with the local legislation and institutional requirements. Written informed consent for participation in this study was provided by the participants' legal guardians/next of kin.

## Author contributions

PR: Conceptualization, Formal analysis, Writing – original draft, Writing – review & editing. AK: Funding acquisition, Methodology, Project administration, Validation, Writing – review & editing. MH: Formal analysis, Software, Visualization, Writing – review & editing. CK: Writing – review & editing. EM: Writing – review & editing. DT: Investigation, Supervision, Writing – review & editing. SL: Investigation, Supervision, Writing – review & editing. TØ: Investigation, Supervision, Writing – review & editing. JP: Supervision, Writing – review & editing. SB: Investigation, Supervision, Writing – review & editing.

## References

[ref1] Ahmedt-Aristizabal D. Armin M. A. Hayder Z. Garcia-Cairasco N. Petersson L. Fookes C.. (2023). Deep learning approaches for seizure video analysis: a review. arXiv Available at: http://arxiv.org/abs/2312.10930 10.1016/j.yebeh.2024.109735 38522192

[ref2] Amin U. Primiani C. T. MacIver S. Rivera-Cruz A. Frontera A. T. Jr. Benbadis S. R. (2021). Value of smartphone videos for diagnosis of seizures: everyone owns half an epilepsy monitoring unit. Epilepsia 62, e135–e139. doi: 10.1111/epi.17001, PMID: 34254664

[ref3] Anne L. (2013). “Chapter 93-psychogenic nonepileptic seizures” in Handbook of clinical neurology [internet]. eds. Dulac O. Lassonde M. Sarnat H. B., vol. 112 (Elsevier), 875–879. (Pediatric Neurology Part II. Available at: https://www.sciencedirect.com/science/article/pii/B978044452910700009X 10.1016/B978-0-444-52910-7.00009-X 23622297

[ref4] Arends J. B. van Dorp J. van Hoek D. Kramer N. van Mierlo P. van der Vorst D.. (2016). Diagnostic accuracy of audio-based seizure detection in patients with severe epilepsy and an intellectual disability. Epilepsy Behav. 62, 180–185. doi: 10.1016/j.yebeh.2016.06.008, PMID: 27490905

[ref5] Armand Larsen S. Terney D. Østerkjerhuus T. Vinding Merinder T. Annala K. Knight A.. (2022). Automated detection of nocturnal motor seizures using an audio-video system. Brain Behav. 12:e2737. doi: 10.1002/brb3.2737, PMID: 35939047 PMC9480955

[ref6] Basnyat P. Mäkinen J. Saarinen J. T. Peltola J. (2022). Clinical utility of a video/audio-based epilepsy monitoring system Nelli. Epilepsy Behav. 133:108804. doi: 10.1016/j.yebeh.2022.108804, PMID: 35753111

[ref7] Beghi E. Giussani G. Nichols E. Abd-Allah F. Abdela J. Abdelalim A.. (2019). Global, regional, and national burden of epilepsy, 1990–2016: a systematic analysis for the global burden of disease study 2016. Lancet Neurol. 18, 357–375. doi: 10.1016/S1474-4422(18)30454-X, PMID: 30773428 PMC6416168

[ref8] Beniczky S. Jeppesen J. (2019). Non-electroencephalography-based seizure detection. Curr. Opin. Neurol. 32, 198–204. doi: 10.1097/WCO.0000000000000658, PMID: 30664069

[ref9] Beniczky S. Rubboli G. Aurlien H. Hirsch L. J. Trinka E. Schomer D. L.. (2017). The new ILAE seizure classification: 63 seizure types? Epilepsia 58, 1298–1300. doi: 10.1111/epi.13799 28677857

[ref10] Beniczky S. Ryvlin P. (2018). Standards for testing and clinical validation of seizure detection devices. Epilepsia 59, 9–13. doi: 10.1111/epi.14049, PMID: 29873827

[ref11] Beniczky S. Wiebe S. Jeppesen J. Tatum W. O. Brazdil M. Wang Y.. (2021). Automated seizure detection using wearable devices: a clinical practice guideline of the international league against epilepsy and the International Federation of Clinical Neurophysiology. Epilepsia 62, 632–646. doi: 10.1111/epi.16818, PMID: 33666944

[ref12] Cattani L. Alinovi D. Ferrari G. Raheli R. Pavlidis E. Spagnoli C.. (2017). Monitoring infants by automatic video processing: a unified approach to motion analysis. Comput. Biol. Med. 80, 158–165. doi: 10.1016/j.compbiomed.2016.11.010, PMID: 27940321

[ref13] Conradsen I. Beniczky S. Hoppe K. Wolf P. Sorensen H. B. D. (2012). Automated algorithm for generalized tonic–Clonic epileptic seizure onset detection based on sEMG zero-crossing rate. I.E.E.E. Trans. Biomed. Eng. 59, 579–585. doi: 10.1109/TBME.2011.2178094, PMID: 22156944

[ref14] Cuppens K. Chen C. W. Wong K. B. Y. Van de Vel A. Lagae L. Ceulemans B.. (2012). “Using spatio-temporal interest points (STIP) for myoclonic jerk detection in nocturnal video” in 2012 annual international conference of the IEEE engineering in medicine and biology society, 4454–4457. 23366916 10.1109/EMBC.2012.6346955

[ref15] Duun-Henriksen J. Baud M. Richardson M. P. Cook M. Kouvas G. Heasman J. M.. (2020). A new era in electroencephalographic monitoring? Subscalp devices for ultra-long-term recordings. Epilepsia 61, 1805–1817. doi: 10.1111/epi.16630, PMID: 32852091

[ref16] Elger C. E. Hoppe C. (2018). Diagnostic challenges in epilepsy: seizure under-reporting and seizure detection. Lancet Neurol. 17, 279–288. doi: 10.1016/S1474-4422(18)30038-3, PMID: 29452687

[ref17] Fazel S. Wolf A. Långström N. Newton C. R. Lichtenstein P. (2013). Premature mortality in epilepsy and the role of psychiatric comorbidity: a total population study. Lancet 382, 1646–1654. doi: 10.1016/S0140-6736(13)60899-5, PMID: 23883699 PMC3899026

[ref18] Fiest K. M. Sauro K. M. Wiebe S. Patten S. B. Kwon C. S. Dykeman J.. (2017). Prevalence and incidence of epilepsy. Neurology 88, 296–303. doi: 10.1212/WNL.0000000000003509, PMID: 27986877 PMC5272794

[ref19] Fisher R. S. Cross J. H. French J. A. Higurashi N. Hirsch E. Jansen F. E.. (2017). Operational classification of seizure types by the international league against epilepsy: position paper of the ILAE Commission for Classification and Terminology. Epilepsia 58, 522–530. doi: 10.1111/epi.13670, PMID: 28276060

[ref20] Garção V. M. Abreu M. Peralta A. R. Bentes C. Fred A. (2023). P. da Silva H. A novel approach to automatic seizure detection using computer vision and independent component analysis. Epilepsia 64, 2472–2483. doi: 10.1111/epi.17677, PMID: 37301976

[ref21] Gavvala J. R. Schuele S. U. (2016). New-onset seizure in adults and adolescents: a review. JAMA 316, 2657–2668. doi: 10.1001/jama.2016.18625 28027373

[ref22] Geertsema E. E. Thijs R. D. Gutter T. Vledder B. Arends J. B. Leijten F. S.. (2018). Automated video-based detection of nocturnal convulsive seizures in a residential care setting. Epilepsia 59, 53–60. doi: 10.1111/epi.14050 29638008

[ref23] Hoppe C. Poepel A. Elger C. E. (2007). Epilepsy: accuracy of patient seizure counts. Arch. Neurol. 64, 1595–1599. doi: 10.1001/archneur.64.11.1595 17998441

[ref24] Johansson D. Ohlsson F. Krýsl D. Rydenhag B. Czarnecki M. Gustafsson N.. (2019). Tonic-clonic seizure detection using accelerometry-based wearable sensors: a prospective, video-EEG controlled study. Seizure 65, 48–54. doi: 10.1016/j.seizure.2018.12.024, PMID: 30611010

[ref25] Kalitzin S. Petkov G. Velis D. Vledder B. (2012). Lopes da Silva F. Automatic segmentation of episodes containing epileptic clonic seizures in video sequences. I.E.E.E. Trans. Biomed. Eng. 59, 3379–3385. doi: 10.1109/TBME.2012.2215609, PMID: 22949042

[ref27] Karácsony T. László J. Fernando D. L. T. F. Cunha J. P. (2023). Deep learning methods for single camera based clinical in-bed movement action recognition. IEEE Available at: https://www.researchgate.net/publication/372799787_Deep_Learning_Methods_for_Single_Camera_Based_Clinical_In-bed_Movement_Action_Recognition

[ref28] Karayiannis N. B. Tao G. Frost J. D. Wise M. S. Hrachovy R. A. Mizrahi E. M. (2006). Automated detection of videotaped neonatal seizures based on motion segmentation methods. Clin. Neurophysiol. 117, 1585–1594. doi: 10.1016/j.clinph.2005.12.030, PMID: 16684619

[ref29] Karayiannis N. B. Tao G. Xiong Y. Sami A. Varughese B. Frost J. D. Jr.. (2005). Computerized motion analysis of videotaped neonatal seizures of epileptic origin. Epilepsia 46, 901–917. doi: 10.1111/j.1528-1167.2005.56504.x, PMID: 15946330

[ref30] Knight A. Gschwind T. Galer P. Worrell G. A. Litt B. Soltesz I.. (2024). Artificial intelligence in epilepsy phenotyping. Epilepsia. 1–14. doi: 10.1111/epi.17833, PMID: 37983589 PMC11102939

[ref31] Kwan P. Arzimanoglou A. Berg A. T. Brodie M. J. Allen Hauser W. Mathern G.. (2010). Definition of drug resistant epilepsy: consensus proposal by the ad hoc task force of the ILAE commission on therapeutic strategies. Epilepsia 51, 1069–1077. doi: 10.1111/j.1528-1167.2009.02397.x, PMID: 19889013

[ref32] LaFrance W. C. Baker G. A. Duncan R. Goldstein L. H. Reuber M. (2013). Minimum requirements for the diagnosis of psychogenic nonepileptic seizures: a staged approach. Epilepsia 54, 2005–2018. doi: 10.1111/epi.12356, PMID: 24111933

[ref33] Laxer K. D. Trinka E. Hirsch L. J. Cendes F. Langfitt J. Delanty N.. (2014). The consequences of refractory epilepsy and its treatment. Epilepsy Behav. 37, 59–70. doi: 10.1016/j.yebeh.2014.05.031 24980390

[ref34] Lee J. W. Khoshbin S. (2008). “CHAPTER 76- Seizure Disorders (Epilepsy)” in Massachusetts General Hospital Comprehensive clinical psychiatry. eds. Stern T. A. Rosenbaum J. F. Fava M. Biederman J. Rauch S. L. (Philadelphia: Mosby), 1041–1052.

[ref35] Lu H. Pan Y. Mandal B. Eng H. L. Guan C. Chan D. W. S. (2013). Quantifying limb movements in epileptic seizures through color-based video analysis. I.E.E.E. Trans. Biomed. Eng. 60, 461–469. doi: 10.1109/TBME.2012.2228649, PMID: 23192478

[ref36] Mehta D. Sivathamboo S. Simpson H. Kwan P. O’Brien T. Ge Z.. Privacy-preserving early detection of epileptic seizures in videos. In: Medical Image Computing and Computer Assisted Intervention–MICCAI 2023-26th International Conference Vancouver, BC, Canada, October 8–12, 2023 Proceedings, Part V [Internet]. Springer; (2023) p. 210–219. Available at: https://research.monash.edu/en/publications/privacy-preserving-early-detection-ofepileptic-seizures-invideos

[ref37] Meritam Larsen P. Wüstenhagen S. Terney D. Gardella E. Aurlien H. Beniczky S. (2023). Duration of epileptic seizure types: a data-driven approach. Epilepsia 64, 469–478. doi: 10.1111/epi.17492, PMID: 36597206 PMC10107943

[ref38] Mielke H. Meissner S. Wagner K. Joos A. Schulze-Bonhage A. (2020). Which seizure elements do patients memorize? A comparison of history and seizure documentation. Epilepsia 61, 1365–1375. doi: 10.1111/epi.16550, PMID: 32515852

[ref39] Milošević M. Van de Vel A. Bonroy B. Ceulemans B. Lagae L. Vanrumste B.. (2016). Automated detection of tonic–Clonic seizures using 3-D Accelerometry and surface electromyography in pediatric patients. IEEE J. Biomed. Health Inform. 20, 1333–1341. doi: 10.1109/JBHI.2015.2462079, PMID: 26241981

[ref40] Naganur V. D. Kusmakar S. Chen Z. Palaniswami M. S. Kwan P. O’Brien T. J. (2019). The utility of an automated and ambulatory device for detecting and differentiating epileptic and psychogenic non-epileptic seizures. Epilepsia Open 4, 309–317. doi: 10.1002/epi4.12327, PMID: 31168498 PMC6546070

[ref41] Ojanen P. Knight A. Hakala A. Bondarchik J. Noachtar S. Peltola J.. (2021). An integrative method to quantitatively detect nocturnal motor seizures. Epilepsy Res. 169:106486. doi: 10.1016/j.eplepsyres.2020.106486, PMID: 33310414

[ref42] Pediaditis M. Tsiknakis M. Leitgeb N. (2012). Vision-based motion detection, analysis and recognition of epileptic seizures--a systematic review. Comput. Methods Prog. Biomed. 108, 1133–1148. doi: 10.1016/j.cmpb.2012.08.005, PMID: 22954620

[ref43] Peltola J. Basnyat P. Armand Larsen S. Østerkjærhuus T. Vinding Merinder T. Terney D.. (2023). Semiautomated classification of nocturnal seizures using video recordings. Epilepsia 64 Suppl 4, S65–S71. doi: 10.1111/epi.17207 35194778

[ref44] Pisani F. Spagnoli C. Pavlidis E. Facini C. Kouamou Ntonfo G. M. Ferrari G.. (2014). Real-time automated detection of clonic seizures in newborns. Clin. Neurophysiol. 125, 1533–1540. doi: 10.1016/j.clinph.2013.12.119, PMID: 24602566

[ref45] Sun Z. Ke Q. Rahmani H. Bennamoun M. Wang G. Liu J. (2022). Human action recognition from various data modalities: a review. IEEE Trans. Pattern Anal. Mach. Intell. 45, 3200–3225. doi: 10.1109/TPAMI.2022.3183112 35700242

[ref46] Surges R. Thijs R. D. Tan H. L. Sander J. W. (2009). Sudden unexpected death in epilepsy: risk factors and potential pathomechanisms. Nat. Rev. Neurol. 5, 492–504. doi: 10.1038/nrneurol.2009.118, PMID: 19668244

[ref47] Sveinsson O. Andersson T. Mattsson P. Carlsson S. Tomson T. (2020). Clinical risk factors in SUDEP: a nationwide population-based case-control study. Neurology 94, e419–e429. doi: 10.1212/WNL.0000000000008741, PMID: 31831600 PMC7079690

[ref48] Szabó C. Á. Morgan L. C. Karkar K. M. Leary L. D. Lie O. V. Girouard M.. (2015). Electromyography-based seizure detector: preliminary results comparing a generalized tonic–clonic seizure detection algorithm to video-EEG recordings. Epilepsia 56, 1432–1437. doi: 10.1111/epi.13083, PMID: 26190150

[ref49] Tang J. El Atrache R. Yu S. Asif U. Jackson M. Roy S.. (2021). Seizure detection using wearable sensors and machine learning: setting a benchmark. Epilepsia 62, 1807–1819. doi: 10.1111/epi.16967, PMID: 34268728 PMC8457135

[ref50] Ulate-Campos A. Coughlin F. Gaínza-Lein M. Fernández I. S. Pearl P. L. Loddenkemper T. (2016). Automated seizure detection systems and their effectiveness for each type of seizure. Seizure 40, 88–101. doi: 10.1016/j.seizure.2016.06.008, PMID: 27376911

[ref51] van Westrhenen A. Petkov G. Kalitzin S. N. Lazeron R. H. C. Thijs R. D. (2020). Automated video-based detection of nocturnal motor seizures in children. Epilepsia 61 Suppl 1, S36–S40. doi: 10.1111/epi.16504, PMID: 32378204 PMC7754425

[ref52] Wolf P. Benbadis S. Dimova P. S. Vinayan K. P. Michaelis R. Reuber M.. (2020). The importance of semiological information based on epileptic seizure history. Epileptic Disord. 22, 15–31. doi: 10.1684/epd.2020.1137 32096471

